# Recent advances in the diagnosis and management of pre-eclampsia

**DOI:** 10.12688/f1000research.12249.1

**Published:** 2018-02-28

**Authors:** Kate Duhig, Brooke Vandermolen, Andrew Shennan

**Affiliations:** 1 Women’s Health Academic Centre, King’s College London, Westminster Bridge Road, London, SE1 7EH, UK

**Keywords:** pre-eclampsia, hypertensive disorders, diagnosis, angiogenic biomarkers, PlGF

## Abstract

Pre-eclampsia is a leading cause of maternal mortality, responsible annually for over 60,000 maternal deaths around the globe. Pre-eclampsia is a multisystem disease featuring hypertension, proteinuria, and renal, hepatic, and neurological involvement. Diagnosis is often elusive, as clinical presentation is highly variable. Even those with severe disease can remain asymptomatic. Angiogenic factors are emerging as having a role in the diagnosis of pre-eclampsia and in prognostication of established disease. In this article, we summarize new developments and focus on angiogenic biomarkers for prediction of disease onset. We also discuss recent advances in management strategies for patients with hypertensive disorders of pregnancy.

## Introduction

Hypertensive disorders of pregnancy are responsible for over 60,000 maternal deaths worldwide annually and complicate 5% of all pregnancies ^1^. Pregnancies complicated by pre-eclampsia show an increase in maternal and perinatal morbidity and mortality.

The definition of pre-eclampsia was revised in 2014 and is defined as hypertension developing after 20 weeks’ gestation with one or more of the following: proteinuria, maternal organ dysfunction (including renal, hepatic, hematological, or neurological complications), or fetal growth restriction ^2^. It is important to note that this definition does not require proteinuria to meet the diagnostic criteria. The inclusion of fetal growth restriction in this definition may increase the number of women meeting the diagnostic criteria for pre-eclampsia and is, therefore, a significant change.

Diagnosing pre-eclampsia remains a challenge. The maternal phenotype of pre-eclampsia is associated with inflammation and endothelial cell activation. The more severe early onset placental phenotype is associated with fetal growth restriction. Women may present with late-onset hypertension and proteinuria, with an absence of fetal growth restriction near term. This appears to have few long-term consequences for mother or infant. Conversely, early onset, severe maternal disease is often associated with fetal intrauterine growth restriction.

Even in the presence of severe preterm disease, a woman can be asymptomatic. Douglas and Redman reported an absence of hypertension and proteinuria in 38% of women who presented with an eclamptic fit ^3^, demonstrating that severe maternal adverse events occur even when the traditional clinical definition of pre-eclampsia is not met. Unrecognized fetal compromise contributes to the rate of fetal demise, and 1 in 20 stillbirths without congenital abnormality is complicated by, or attributable to, pre-eclampsia ^4^.

## New developments in prediction

An important focus for improving the antenatal management of pre-eclampsia is to develop accurate prediction models that identify women at high risk of disease. This would enable more appropriate targeting of prophylaxis from the first trimester as well as increased surveillance of those at high risk of disease. Lack of recognition of risk contributes to substandard care associated with maternal deaths ^5^. Early administration of prophylactic aspirin in high-risk women prior to 16 weeks’ gestation appears to reduce the risk of pre-eclampsia by 17%. Furthermore, there is an 8% relative risk reduction of preterm birth and a 14% reduction in fetal and neonatal death ^6^.

### Risk factors

The National Institute for Health and Care Excellence (NICE) recommends a list of maternal risk factors that can be used to identify women at high risk for pre-eclampsia in whom aspirin should be started from 12 weeks’ gestation ^7^. Strong risk factors include previous pre-eclampsia or hypertension in pregnancy, chronic kidney disease, chronic hypertension, diabetes (type 1 or 2), and autoimmune disorders such as systemic lupus erythematosus or antiphospholipid syndrome. Women should also be advised to take aspirin if they have more than one of the following moderate risk factors: first pregnancy, age of 40 years or more, a pregnancy interval of greater than 10 years, body mass index of 35 kg/m ^2^ or more, family history of pre-eclampsia, and multiple pregnancy ^8^. A recent study demonstrated that women who developed pre-eclampsia had a significantly lower vitamin D concentration at 14 weeks compared with women in the control group (mean 47.2 versus 52.3 nmol/L, *p*<0.0001) ^9^. Women with levels below 30 nmol/L compared with those with at least 50 nmol/L had a greater risk of developing pre-eclampsia—adjusted odds ratio (OR) 2.23; 95% confidence interval (CI) 1.29–3.83—after adjustment for other variables, suggesting that maternal vitamin D deficiency may be an independent risk factor for the development of pre-eclampsia. Indeed, the large SCOPE (Screening for Pregnancy Endpoints) cohort study ^10^ showed that there was a lower incidence of developing pre-eclampsia with a small-for-gestational-age baby with 25(OH)D concentrations of more than 75 nmol/L at 15 weeks’ gestation. Another large prospective cohort study from New Zealand failed to demonstrate similar findings ^11^. To accurately investigate this link requires well-designed randomized controlled studies.

Low dietary calcium and low serum calcium concentrations are associated with pre-eclampsia. It has been shown that high-dose calcium supplementation reduces pre-eclampsia in women from areas with low dietary calcium intake (relative risk 0.36, 95% CI 0.23–0.57) ^12^. Although calcium supplementation is not recommended in women with normal dietary calcium intake, the World Health Organization recommends calcium supplementation (1.5–2 g daily) in the second half of pregnancy for women with low dietary calcium intake. A randomized controlled trial investigating the use of early low-dose dietary calcium supplementation in women who have had previously developed pre-eclampsia did not demonstrate a significant benefit in reduction of blood pressure or subsequent risk of pre-eclampsia ^13^.

A novel “point of care” test used for the prediction of pre-eclampsia is the measurement of glycosylated fibronectin (GlyFn) serum levels in the first trimester. A longitudinal cohort study by Rasanen *et al*. showed levels to be significantly higher in women with pre-eclampsia ( *p*<0.01) and to remain higher throughout pregnancy ( *p*<0.01) ^14^. Increased GlyFn levels were significantly associated with blood pressure and small-for-gestational-age neonates. Analysis of measurements in mild pre-eclampsia showed a weekly change of 81.7 mg/mL (standard error [SE] 94.1) versus 195.2 mg/mL (SE 88.2) for severe pre-eclampsia. It remains to be seen whether predictive ability has sufficient utility to add to risk prediction in clinical practice.

### Risk modeling

Pre-eclampsia is notoriously difficult to predict and diagnose. There have been many studies investigating multiple-marker algorithms for predicting pre-eclampsia in a manner similar to that already in use at first-trimester aneuploidy screening. Significant differences have been shown in mean first-trimester levels of pregnancy-associated para protein A (PAPP-A), a disintegrin and metalloproteinase 12 (ADAM12), and placental growth factor (PlGF) ^15^; placental protein 13 ^16^; angiopoietin 1 and 2 ^17^; inhibin A and Activin A, soluble endoglin, and soluble fms-like tyrosine kinase 1 (sFlt-1) ^18^; and human chorionic gonadotropin (hCG) ^19^. However, alone and in combination, their likelihood ratios have been insufficient for use as reliable prognostic tools for pre-eclampsia. This was demonstrated in a systematic review into the methodology of studies developing first-trimester prediction models. The authors reported frequent methodological deficiencies in studies reporting risk prediction models for pre-eclampsia, which may limit their reliability and validity ^20^.

Several studies have shown that levels of cell-free fetal DNA (cffDNA) are raised in women with pre-eclampsia ^21, 22^. The hypothesis for increased levels of cffDNA is of abnormal placentation, hypoxia reperfusion injury, and release of apoptotic fragments containing cffDNA into maternal circulation ^22^. A recent systematic review showed that whilst cffDNA may have a role in disease prediction in pre-eclampsia, its use is probably limited to the early second trimester because its detection rate is too low at later gestations ^23^.

A further systematic review compared “simple” risk models for pre-eclampsia that use routinely collected maternal characteristics against “specialized” models that include specialized tests ^24^. Four simple models were externally validated, and a model using parity, previous pre-eclampsia, race, chronic hypertension, and conception method to predict early onset pre-eclampsia achieved the highest area under the curve (AUC) (0.76, 95% CI 0.74–0.77). Nine studies comparing simple versus specialized models in the same population reported AUCs favoring specialized models. However, a simple model achieved fewer false positives than a guideline-recommended risk factor list such as NICE hypertension-in-pregnancy guideline, but sensitivity to classify risk for aspirin prophylaxis was not assessed.

### Assessing pre-eclampsia

Pre-eclampsia is elusive to diagnose. Hypertension is classified as a blood pressure of at least 140/90 mmHg. Those with a background of chronic hypertension are at higher risk of developing pre-eclampsia and remain a challenge to diagnose, as conventional blood pressure thresholds are not always applicable.

There is evidence of accuracy, increased surveillance, and acceptability of home blood pressure monitoring in pregnancy in small studies ^25– 27^. However, a systematic review of ambulatory versus conventional monitoring of blood pressure in pregnancy found no evidence to support its routine use ^28^. OPTIMUM (optimizing titration and monitoring of maternal blood pressure) is an ongoing randomized controlled study assigning women with high blood pressure to self-monitoring in addition to antenatal care versus usual antenatal care to identify rising blood pressure sooner, which could lead to an earlier diagnosis and treatment of subsequent complications ^29^. Also under way is the BUMP trial (Blood Pressure monitoring in High-Risk Pregnancy to Improve the Detection and Monitoring of Hypertension). This randomized controlled trial compares routine antenatal care with self-monitoring in high-risk women to determine whether self-monitoring can lead to earlier diagnosis of hypertension and lower blood pressure in those with hypertension and pre-eclampsia ^30^.

Additionally, assessment of proteinuria is variable. Even this gold standard, defined as greater than 300 mg of protein excreted in the urine in 24 hours, is prone to error ^31^. There is heterogeneity in test accuracy of protein/creatinine ratios when compared with 24-hour urine collections in pregnancy ^32^. A systematic review and diagnostic meta-analysis suggested that a 12-hour urine collection performs well for the diagnosis of proteinuria in hypertensive women during pregnancy ^32^ and recommends a cut-off of 150 mg per 12-hour collection. This cut-off is associated with 99% specificity and 92% sensitivity for the diagnosis of pre-eclampsia. The use of the 12-hour urine collection would be more convenient, expedite diagnosis and clinical management, and decrease cost ^33^. The results are awaited of the “diagnostic accuracy in pre-eclampsia using proteinuria assessment” (DAPPA) study, aimed at comparing the diagnostic accuracy of different methods of assessing proteinuria at various thresholds in predicting severe pre-eclampsia compared with 24-hour urine protein measurement ^34^.

### Novel methods of diagnosis

**Figure 1.**
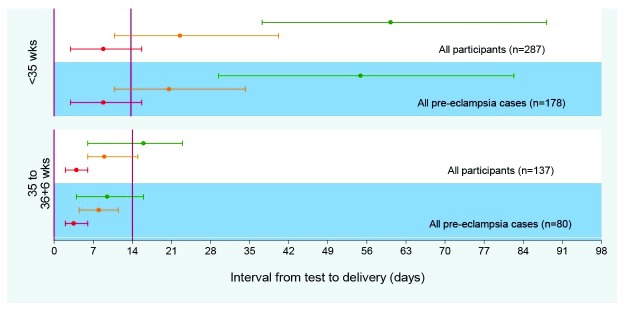
Time to delivery (median, interquartile range) stratified by PlGF concentration for all participants and for pre-eclampsia cases ^28^. Red line indicates very low PlGF (<12 pg/mL); orange line, low PlGF (< fifth centile); green line, normal PlGF (≥ fifth centile). PlGF, placental growth factor.

A role for angiogenic biomarkers in the diagnosis of pre-eclampsia is emerging. Currently, diagnosis relies on parameters associated with end-organ complications of established disease. Angiogenic factors are implicated in the pathophysiology of pre-eclampsia, which may have the potential of identifying women earlier in their disease course. Low maternal PlGF concentrations (defined as below the fifth centile for gestation or not more than 100 pg/mL) have demonstrated high sensitivity (0.96, 95% CI 0.89–0.99) and a negative predictive value (0.98, 95% CI 0.93–0.995) for predicting the development of pre-eclampsia that requires delivery within 14 days ( Figure 1) ^35^. These very low PlGF concentrations were often seen weeks prior to the diagnosis of pre-eclampsia in this cohort. “Prediction of short-term outcome in pregnant women with suspected pre-eclampsia” (PROGNOSIS) by Zeisler *et al*., a prospective, multicenter observational study of 500 women, demonstrated that a sFlt-1:PlGF ratio cut-off of 38 has clinical utility ^36^. Values below this cut-off have a high negative predictive value (99.3%, 95% CI 97.9–99.9) and an 80% sensitivity (95% CI 51.9–95.7) and 78.3% specificity (95% CI 74.6–81.7) for pre-eclampsia. The positive predictive value of an sFlt-1:PlGF ratio above 38 for a diagnosis of pre-eclampsia within 4 weeks was 36.7% (95% CI 28.4–45.7), sensitivity was 66.2% (95% CI 54.0–77.0), and specificity was 83.1% (95% CI 79.4–86.3). The authors propose that in women in whom pre-eclampsia is suspected clinically, an sFlt-1:PLGF ratio of less than 38 can be used to rule out the short-term development of the syndrome.

The most likely area of clinical impact for PlGF is in “point-of-care” testing in women posing a diagnostic challenge to the clinician. These “point-of-care” tests could have a substantial impact on health resource use, avoiding unnecessary admissions for those who will have a more benign disease course and a longer “time to delivery” interval. A cost-saving analysis performed in 2010 showed that the addition of an angiogenic biomarker test can amount to a saving of £945 per woman because of its ability to reduce the rates of false-positive and false-negative diagnoses compared with the current standard of care ^37^. In 2016, a budget impact analysis in consultant-led maternity units modeled that PlGF testing was associated with a mean cost saving of £582 per woman tested ^38^. A similar health economic assessment published in the same year demonstrated a £344 cost saving per patient ^39^. Such tests have the potential to assist in risk stratification in women at high risk of developing pre-eclampsia, singling out those with low PlGF to receive intensive surveillance to avoid adverse outcomes such as fetal demise. The “placental growth factor to assess and diagnose hypertensive pregnant women: a stepped wedge trial” (PARROT) is under way to determine whether the addition of PlGF testing to the current management of women with pre-eclampsia will reduce the time taken to reach diagnosis and thus improve maternal and perinatal outcomes ^40^.

Placental exosomes have been highlighted for use in the diagnosis of pre-eclampsia. They are extracellular vesicles which can transfer microRNA to target cells, influencing their function ^41^. There is evidence to show abnormal levels of circulating microRNAs in pregnancies affected by pre-eclampsia ^42– 44^. In a recent prospective cohort, it was shown that the levels of circulating exosomes are increased in pregnancies complicated by pre-eclampsia and that this difference is seen across gestations ^45^. The maintenance of this difference across gestations could suggest a potential role for exosomal microRNA in both the prediction and the diagnosis of pre-eclampsia.

In practice, it is important to consider that women classified with a “low-risk” pregnancy at booking still need a full antenatal care schedule, including frequent assessment to exclude hypertension and proteinuria.

## Management

### Blood pressure

The NICE recommends keeping systolic blood pressure below 150 mmHg and diastolic blood pressure below 80–100 mmHg ^7^ and using labetalol as first-line treatment for hypertension over this threshold. The results of the Control of Hypertension In Pregnancy Study (CHIPS) were reported in 2016. This trial compared “tight” (target diastolic blood pressure of 85 mmHg) versus “less tight” (target diastolic blood pressure of 100 mmHg) control of hypertension in women with non-severe, non-proteinuric maternal hypertension at 14–33 weeks ^46^. The results demonstrated that those with “tight” control achieved a lower blood pressure (by 5 mmHg) and there was no increase in adverse perinatal outcome (adjusted OR 0.98, 95% CI 0.74–1.3) and birth weight less than the tenth percentile (1.3, 0.93–1.8). However, there were reduced rates of severe maternal hypertension ( *p*<0.001) with tighter control. In this trial, 48.9% of the women developed pre-eclampsia in the “less tight” group and 45.7% in the “tight” control group (adjusted OR 1.14, CI 0.88–1.47). While results from this study can only be extrapolated to pre-eclampsia with caution, it may be concluded that in these women who are at high risk of the complications of severe hypertension, seizures, and intracerebral hemorrhage, there may be benefit in tighter control of blood pressure.

### Oral antihypertensives

Traditionally, severe hypertension has been treated with short-acting parenteral antihypertensive agents, most frequently intravenous hydralazine or labetalol. This is because of the speed of onset of action but means that they require more intensive monitoring and can affect the fetus if large shifts in blood pressure occur. A systematic review showed that, in most women, nifedipine achieved treatment success similar to that of hydralazine (84% with nifedipine; relative risk 1.07, 95% CI 0.98–1.17) or labetalol (100% with nifedipine; relative risk 1.02, 95% CI 0.95–1.09) ^47^. Less than 2% of women who received nifedipine experienced hypotension. There were no differences in adverse maternal or fetal outcomes. Thus, the authors suggest that oral nifedipine is a suitable treatment for severe hypertension in pregnancy and post-partum.

A meta-analysis by Shekhar *et al*. confirmed these findings, providing further evidence that oral nifedipine is a reasonable antihypertensive for the treatment of severe pregnancy hypertension of any classification ^48^. These treatments are widely available, even in middle- and lower-income countries, so these findings can be implemented globally and reduce costs.

### Delivery

Clinical convention offers women with pre-eclampsia delivery at 37 weeks’ gestation as per NICE guidance and the guideline from the American College of Obstetricians and Gynecologists. Prior to 34 weeks’ gestation, management is expectant with elective delivery not considered due to worse neonatal adverse outcomes (respiratory distress syndrome risk ratio 2.3, 95% CI 1.39–3.81 and necrotizing enterocolitis risk ratio 5.54, 95% CI 1.04–29.56) ^49^. Between 34 and 37 weeks’ gestation, the optimum time to deliver to prevent morbidity for the mother and baby remains unknown.

The HYPITAT-II randomized controlled trial investigated the effect of immediate delivery versus expectant management between 34 and 37 weeks’ gestation in women with non-severe hypertensive disorder of pregnancy including, but not limited to, pre-eclampsia ^50^. The findings of the trial showed that in women diagnosed between 34 and 37 weeks of gestation, immediate delivery (through either induction or, if indicated, elective cesarean section) might reduce the small risk of adverse maternal outcomes compared with expectant monitoring (assessed by a composite of severe maternal adverse outcomes; relative risk 0.36, 95% CI 0.12–1.11; *p*=0.067). However, immediate delivery may increase the risk of neonatal respiratory distress syndrome (relative risk 3.3, 95% CI 1.4–8.2; *p*=0.005). It is important to note that this trial was not specifically powered for neonatal endpoints and still showed a clinically important (70%) non-significant benefit to the mother; therefore, further evidence is required before making firm recommendations in management. The PHOENIX (pre-eclampsia in hospital: early induction or expectant management) randomized controlled trial is under way to determine whether planned delivery between 34+0 and 36+6 weeks of gestation in women with pre-eclampsia reduces adverse maternal outcomes without substantially increasing neonatal/infant morbidity ^51^.

### Complications

A diagnosis of pre-eclampsia may result in a range of complications with significant long-term implications for the mother ^52^. Furthermore, the diagnosis may have future implications for the management of the health of the mother, as a history of pre-eclampsia is an independent risk factor for cardiac events and stroke. Women from the HYPITAT trial, which investigated the optimum time for delivery in women with gestational hypertension or pre-eclampsia, received a cardiovascular follow-up 2–5 years post-delivery. The results showed that almost half of the early onset pre-eclampsia women subsequently developed hypertension as opposed to 39% and 25% of women in the pregnancy-induced hypertension and late-onset pre-eclampsia groups, respectively ^53^. The effects can be even longer-lasting, and it has been shown that 30 years after a pregnancy complicated by pre-eclampsia, the odds of having a coronary artery calcification score of greater than 50 Agatston units are 2.61 (CI 0.95–7.14) times greater than those for women without pre-eclampsia, even after adjustment for additional risk factors ^54^.

## Conclusions

Important evidence regarding the optimum methods of diagnosis and management of this complex disease is still emerging. The CHIPS trial demonstrates the importance of optimal management of blood pressure in pregnancy hypertension. In order for optimal management to be instigated, improved diagnosis and surveillance of pre-eclampsia are key. Angiogenic biomarkers demonstrate a promising role for the prediction and diagnosis of pre-eclampsia. There may be a role for PlGF in the monitoring and prognosis of established disease; however, until clinical management and interventions such as timing of delivery are more evidence based, their biggest impact will remain in women presenting with suspected disease as a point-of-care test.

The most serious of all complications of pre-eclampsia is maternal death. The recent MBRACE (mothers and babies: reducing risk through audits and confidential enquiries across the UK) report has demonstrated that UK maternal deaths from hypertensive disorders are at the lowest rate ever ^55^. Investigators found that in 93% of the cases of women who died, there were improvements that could have been made in clinical care, showing that work remains to optimize management for all women. It is, however, a triumph of modern obstetrics that, per million births, there is fewer than one maternal death from hypertensive disorders of pregnancy.

## Abbreviations

AUC, area under the curve; cffDNA, cell-free fetal DNA; CHIPS, Control of Hypertension In Pregnancy Study; CI, confidence interval; GlyFn, glycosylated fibronectin; NICE, National Institute for Health and Care Excellence; OR, odds ratio; PlGF, placental growth factor; SE, standard error; sFlt-1, soluble fms-like tyrosine kinase 1.
